# Relationship between Plasma Pituitary Adenylate Cyclase-Activating Polypeptide (PACAP) Level and Proteome Profile of Cows

**DOI:** 10.3390/ani12121559

**Published:** 2022-06-16

**Authors:** Levente Czegledi, Eva Csosz, Gabriella Gulyas

**Affiliations:** 1 Department of Animal Science, Faculty of Agricultural and Food Sciences and Environmental Management, Institute of Animal Science, Biotechnology and Nature Conservation, University of Debrecen, 138 Boszormenyi Street, 4032 Debrecen, Hungary; czegledi@agr.unideb.hu; 2 Department of Biochemistry and Molecular Biology, Faculty of Medicine, University of Debrecen, 98 Nagyerdei Square, 4032 Debrecen, Hungary; cseva@med.unideb.hu

**Keywords:** PACAP, cattle, protein expression, 2D-PAGE

## Abstract

**Simple Summary:**

Molecular mechanisms underlying the production of livestock are of great interest at all times. Pituitary adenylate cyclase-activating polypeptide (PACAP) is a multifunctional neuropeptide; it takes part in the regulation of various physiological processes, such as feeding, reproduction, thermoregulation and brain development. This study was conducted to investigate the effect of high plasma PACAP levels and low plasma PACAP levels on the protein composition of blood plasma. Our finding is that plasma PACAP level is associated with the abundance of 10 plasma proteins. The functions of the differentially expressed proteins indicate that the PACAP level of plasma is related with the lipid metabolism and immune status of cattle.

**Abstract:**

Pituitary adenylate cyclase-activating polypeptide (PACAP) is a pleiotropic and multifunctional neuropeptide; it takes part in the regulation of various physiological processes, such as feeding, reproduction, catecholamine synthesis, thermoregulation, motor activity, brain development and neuronal survival. Since PACAP plays important regulatory roles, we hypothesized that the level of PACAP in blood is associated with expression of other proteins, which are involved in different metabolic pathways. The objective of the present study was to compare plasma protein profiles of cows with high and low plasma PACAP levels. Differential proteome analyses were performed by two-dimensional gel electrophoresis (2D-PAGE) followed by tryptic digestion and protein identification by liquid chromatography–mass spectrometry (LC-MS). A total of 210 protein spots were detected, and 16 protein spots showed statistically significant differences (*p* < 0.05) in the expression levels between groups. Ten spots showed a higher intensity in the high-PACAP-concentration group, while six spots were more abundant in the low-PACAP-concentration group. The functions of the differentially expressed proteins indicate that the PACAP level of plasma is related to the lipid metabolism and immune status of cattle.

## 1. Introduction

Pituitary adenylate cyclase-activating polypeptide (PACAP) is a pleiotropic and multifunctional neuropeptide and a member of the vasoactive intestinal peptide (VIP)/secretin/glucagon peptide family [1]. Neuropeptides are signalling molecules in the central nervous system, and on the periphery, are involved in the physiological functions, acting as hormones, neurotransmitters or neuromodulators. In the last 30 years, several functions of the peptide have been described; it takes part in the regulation of various physiological processes, such as feeding, reproduction, catecholamine synthesis, thermoregulation, motor activity, brain development and neuronal survival [2].

PACAP is found in two bioactive forms, PACAP27 and PACAP38, of which PACAP38 is the major form in mammalian tissues [3]. The PACAP molecule appears to have been highly conserved in vertebrates and shows high homology to VIP [4]. Both peptides bind to two common G protein-coupled receptors (GPCRs), VPAC1-R and VPAC2-R (vasoactive intestinal polypeptide receptor 1, 2), while PACAP has an additional specific receptor, PAC1-R (pituitary andenylate cyclase 1 receptor) [5]. One of the most studied effects of PACAP is its endogenous protective effect due to anti-apoptotic, anti-inflammatory and antioxidant actions [6]. In the tissues of the central nervous system, the highest concentration of PACAP was measured, but the peptide has been found in other organs, such as the pancreas, testis, gastrointestinal tract, hypophysis and adrenal medulla, as well [3].

Only a few papers have been published so far on the presence and functions of pituitary adenylate cyclase-activating polypeptide in livestock species [7,8,9,10,11]. Czegledi and his collegaues [10] have observed that PACAP was present in the plasma and the milk of sheep, goat and cattle in a similar concentration to that measured in humans. There is no clear evidence for the source of PACAP in bovine plasma [12]. It could originate from sensory nerve endings or from the placenta [13,14]. The source of PACAP in milk may arise from blood plasma or from sensory and autonomic nerve endings as in the case of plasma [15]. Tamas et al. [16] hypothesized that PACAP in milk may be important for the growth and development of newborns, for the development of the immune system and immunological microenvironment of the gastrointestinal tract and for the growth and function of the mammary gland, and thereby the lactation. Since PACAP plays important regulatory roles in different physiological processes, we hypothesized that the level of PACAP in blood is associated with expression of other proteins, which are involved in different metabolic pathways. The association of PACAP expression, through its presence in blood and plasma molecular factors, may reveal a deeper knowledge on its biochemical and physiological function in lactating dairy cows. The differences in blood plasma proteome may cause alteration in milk properties and production. 

The objective of the present study was to compare plasma protein profiles of cows with high and low plasma PACAP levels. 

## 2. Materials and Methods

### 2.1. Animals and Sampling

A total of 74 Holstein cows were included in the study for blood sampling, and 10 of them were used for further analysis. All cows were in their second or third lactation, and pregnant cows were excluded from the study. Cows were housed at a conventional dairy farm and fed with corn silage supplemented with concentrate. 

Blood sample was collected from the tail vein, placed in a sterile 9 mL blood tube containing 150 µL of protease inhibitor aprotinin (200 mg/mL, Trasylol, Bayer) and cooled and centrifuged at 2000 rpm for 10 min at 4 °C. Plasma was removed from each sample, divided into aliquots and immediately frozen at −80 °C until further analysis. 

Study was conducted according to the guidelines imposed by the European Union Directive 2010/63/EU on the protection of animals used for scientific purposes.

### 2.2. Determination of Plasma PACAP Concentration

Bovine PACAP enzyme-linked immunosorbent assay kit (NeoBioLab, Cambridge, MA, USA) was used to measure the plasma PACAP concentration. Assay was performed according to the manufacturer’s instructions. Plasma samples were diluted 100 times, and all samples and standards were measured in duplicate. Absorbance data were collected with a SPECTROstar Nano (BMG Labtech, Ortenberg, Germany) microplate reader set to 450 nm, and then raw ODs were corrected by subtraction of absorbance measured at 540 nm. Sample concentrations were interpolated from a standard curve created by four-parameter logistic curve fit using MARS data analysis software 3.10 (BMG Labtech, Ortenberg, Germany). PACAP level of 74 cows was determined, and 5 cows with plasma PACAP level below 100 pg/mL were grouped into the low-PACAP-level group (mean: 63.02 pg/mL, SEM: 5.86) and 5 cows above 500 pg/mL were allocated to the high-PACAP-level group (mean: 657.64 pg/mL, SEM: 35.16).

### 2.3. Extraction of Proteins

Plasma samples, containing up to 500 µg of protein in a final volume of 100 µL, were precipitated following the protocol provided with ReadyPrep 2-D Cleanup Kit (Bio-Rad, Hercules, CA, USA). The procedure works by quantitatively precipitating and concentrating proteins in a sample while leaving behind and washing away substances such as ionic detergents, nucleic acids, lipids and salts, all of which are known to interfere with IEF. After precipitation, the protein pellets were washed and then resuspended in rehydration buffer (7 M urea, 2 M thiourea, 2% (*w*/*v*) CHAPS, 40 mM DTT, 0.2% (*v*/*v*) Bio-Lyte 4/6 and 6/8 ampholyte at a ratio of 1:2). The protein concentration was determined using RC DC protein assay kit (Bio-Rad, Hercules, CA, USA) with bovine serum albumin (BSA) as standard. The protein concentration of the samples was in the range of 4.2–4.8 mg/mL.

### 2.4. Two-Dimensional Polyacrylamide Gel Electrophoresis (2D-PAGE)

For the first dimension (isoelectric focusing) of two-dimensional gel electrophoresis, 7 cm immobilized pH gradient strips (pH 5–8, linear, (Bio-Rad, Hercules, CA, USA) were rehydrated by passive rehydration using samples dissolved in 125 µL of rehydration buffer (2 M thiourea, 7 M urea, 2% (*w*/*v*) CHAPS, 40 mM DTT, 0.2% (*v*/*v*) Bio-Lyte 4/6 and 6/8 ampholyte at a ratio 1:2, 0.002% (*w*/*v*) bromophenol blue) for 16 h at room temperature. A total of 200 µg of protein was loaded, and the isoelectric focusing was conducted in Protean IEF Cell (Bio-Rad, Hercules, CA, USA). Low voltage (250 V) was applied for 20 min, and then the voltage was gradually increased to 4000 V over 2.5 h and maintained at that level until a total of 20,000 Vh was reached. The current limit was adjusted to 50 mA per strip, and the run was carried out at 20 °C. Focused IPG strips were equilibrated for 10 min in 6 M urea, 20% (*v*/*v*) glycerol, 2% (*w*/*v*) SDS, 50 mM Tris pH 8.8 and 2% (*w*/*v*) DTT, and then for an additional 10 min in the same buffer with the exception that DTT was replaced by 2.5% (*w*/*v*) iodoacetamide. After equilibration, proteins were separated in the second dimension using OmniPAGE Mini (Cleaver Scientific, Rugby, UK) vertical electrophoresis system. Second dimension was performed on 100 × 100 mm, 13% polyacrylamide gels (37.5:1 acrylamide:bis-acrylamide ratio). The gels were run by applying 80 V in the first 10 min and then 180 V until the bromophenol blue dye marker reached the end of the gels. A cooling system provided a constant running temperature of 20 °C. Polyacrylamide gels were stained with colloidal coomassie G-250 (Thermo Fisher Scientific, Waltham, MA, USA) overnight [17]. 2D-PAGE analysis was carried out in three technical replicates of each biologically independent sample; consequently, 15 gels per group, a total of 30 gels, were analyzed.

### 2.5. Image and Data Analysis

Polyacrylamide gel images were scanned using PharosFX Plus (Bio-Rad, Hercules, CA, USA) fluorescent system, and images were analysed using Delta2D software (Decodon™ GmbH, Greifswald, Germany). For gel analysis, all gel images within a group were warped to the first gel image. Spots were automatically detected, and images were checked by eye for undetected or incorrectly detected spots. A reference gel was created by fusing all images using union fusion. Every spot on each gel was quantified and normalized according to the total intensity of all spots on each gel. Student’s *t*-test was performed to assess the statistical significance of spot volumes at 95% confidence level (*t*-test; *p* < 0.05). 

Principal component analysis (PCA) was performed on spot volumes with statistically significant (*p* < 0.05) intensity difference between the low- and high-PACAP-concentration groups using GraphPad Prism 9.3.1. The optimal number of PCs was found to be 2. The loading plot of PC1 and PC2 was examined to reveal the spot differences between the low- and high-PACAP-concentration groups.

For subsequent mass spectrometric analysis, coordinates of spots that showed statistically significant differences were transferred to a preparative gel for spot picking. 

### 2.6. Protein Identification

Protein identification was carried out in the University of Debrecen Proteomics Core Facility. The spots were manually cut out from the gel in a laminar flow cabinet to prevent keratin contamination and destained by washing with 50% (*v*/*v*) acetonitrile in 25 mM ammonium bicarbonate solution. Trypsin digestion was carried out using stabilized MS grade bovine trypsin (12.5 ng/µL) at 37 °C overnight. Peptides were extracted with 5% (*v*/*v*) formic acid, and washed twice with 60% (*v*/*v*) acetonitrile in 1% formic acid. The digested peptides were concentrated with speed-vac and redissolved in 10 µL of 1% formic acid (*v*/*v*). 

The LC-MS/MS analysis was performed on a 4000 QTRAP (AB Sciex, Framingham, MA, USA) mass spectrometer coupled to an Easy nLC II nanoHPLC (Bruker, Billerica, MA, USA). The peptides were purified on a Zorbax 300SB-C18 precolumn (Agilent, Santa Clara, CA, USA) and separated on a reverse-phase Zorbax 300SB-C18 analytical column (Agilent, Santa Clara, CA, USA) using a 90 min water–acetonitrile gradient and 300 nL/min flow rate. Information-dependent acquisition was performed, and the two most intensive ions were used for MS/MS. During analyses, the spray voltage was 2800 V, the nebulizing gas was 50 psi, the curtain gas was 10 psi, the source temperature was 70 °C, and the declustering potential was 50 V.

The acquired LC-MS/MS spectra were used for protein identification; the ProteinPilot 4.5 (AB Sciex, Framingham, MA, USA) search engine, the Uniprot/SwissProt database with no species restriction, trypsin as the modifying enzyme and the biological modification table included in the ProteinPilot 4.5 were managed. For protein identification, a minimum of two peptides with more than 95% confidence was required.

### 2.7. Network Analysis

Proteins identified in spots with statistically significant changes between the groups, plus PACAP, were included in a network analysis. For network generation, the String database was used (https://string-db.org/ accessed on 6 June 2021), and a maximum of 50 first-shell interactors of the query proteins were used under maximum stringency (0.9). The networks and the enriched gene ontology (GO) functions were examined. Two networks were generated, one for each studied group. The bovine immunoglobulins were not found in the String database, so they were omitted from the analyses.

## 3. Results

**Figure 1:**
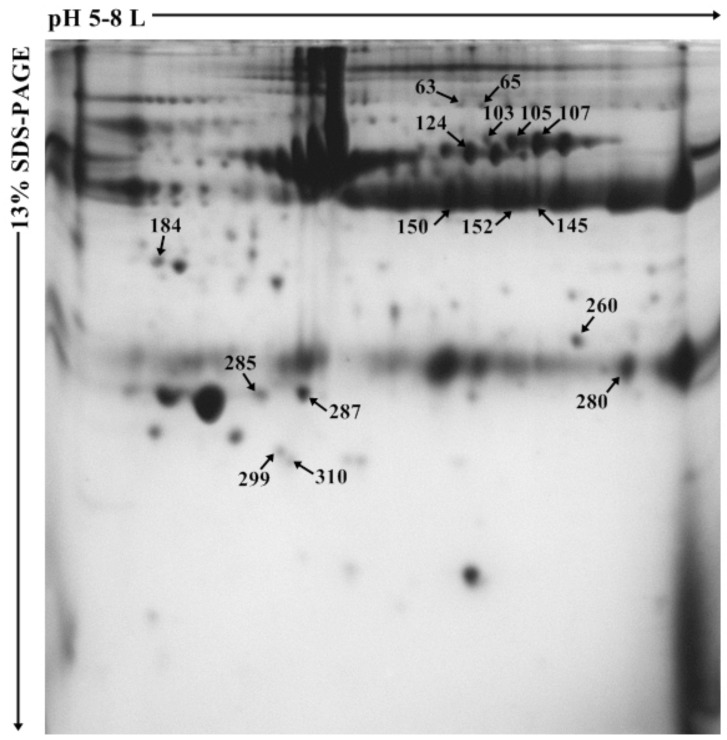
Representative 2D–PAGE image of bovine plasma. Spots with statistically significant intensity difference (*p* < 0.05) between low– and high–PACAP (pituitary adenylate cyclase-activating polypeptide) -concentration groups are marked with numbers.

Bovine plasma samples belonging to different plasma PACAP level groups were analysed by two-dimensional gel electrophoresis followed by mass spectrometric protein identification. As the result of 2D-PAGE, on average 210 protein spots were detected on each gel. Sixteen protein spots showed statistically significant differences (*p* < 0.05) in the expression levels between the high- and low-PACAP-concentration groups (Figure 1). 

**Figure 2:**
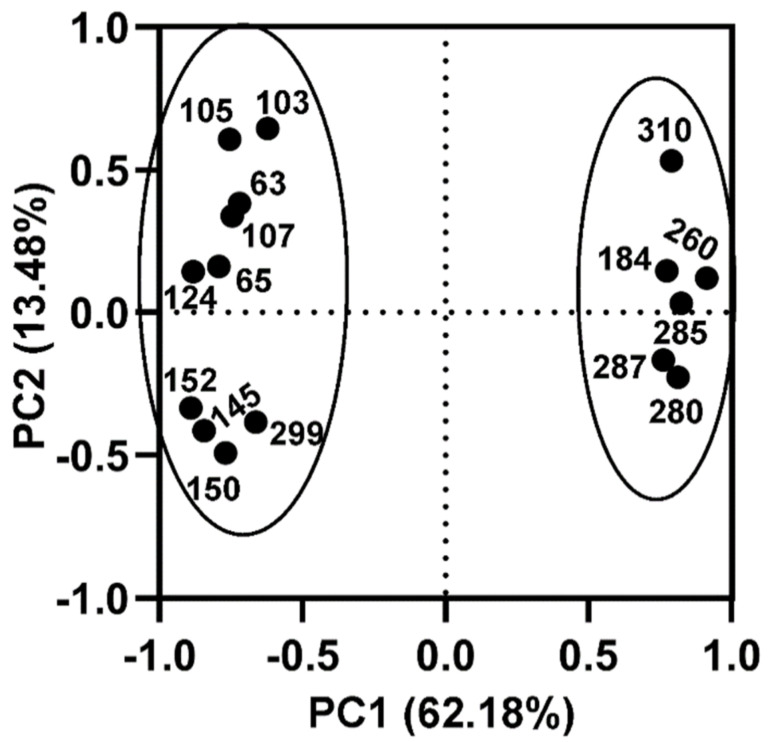
Principal component analysis on spot volume data with statistically significant (*p* < 0.05) intensity difference between the low− and high−plasma-PACAP (pituitary adenylate cyclase-activating polypeptide) −level cows. The two principal components (PC1 and PC2) explaining the majority of the variation in the dataset are plotted against each other. This plot explains 62.18% + 13.48% = 75.65% of the variation in the data.

Principal component analysis (PCA) was performed on spot volumes with statistically significant (*p* < 0.05) intensity differences between the low- and high-PACAP-concentration groups. PCA is a statistical method for determination of the key variables in a multidimensional dataset that explains the differences in the observations. Individual principal component ‘loadings’ represent the contribution of individual protein spot volumes to the variation in the data. This enables the demonstration of the relative contribution of these proteins to proteome differences present between high-PACAP and low-PACAP-plasma-level cows, as well as the identification of clusters of proteins that behave similarly. Protein spots with higher intensities in one of the cow groups based on their PACAP plasma levels appear in the same cluster of the PCA plot. For example, spots 184, 260, 280, 285, 287 and 310 all showed higher volumes in the low-PACAP-level group, and loadings are close to each other on the PCA plot (Figure 2).

**Figure 3:**
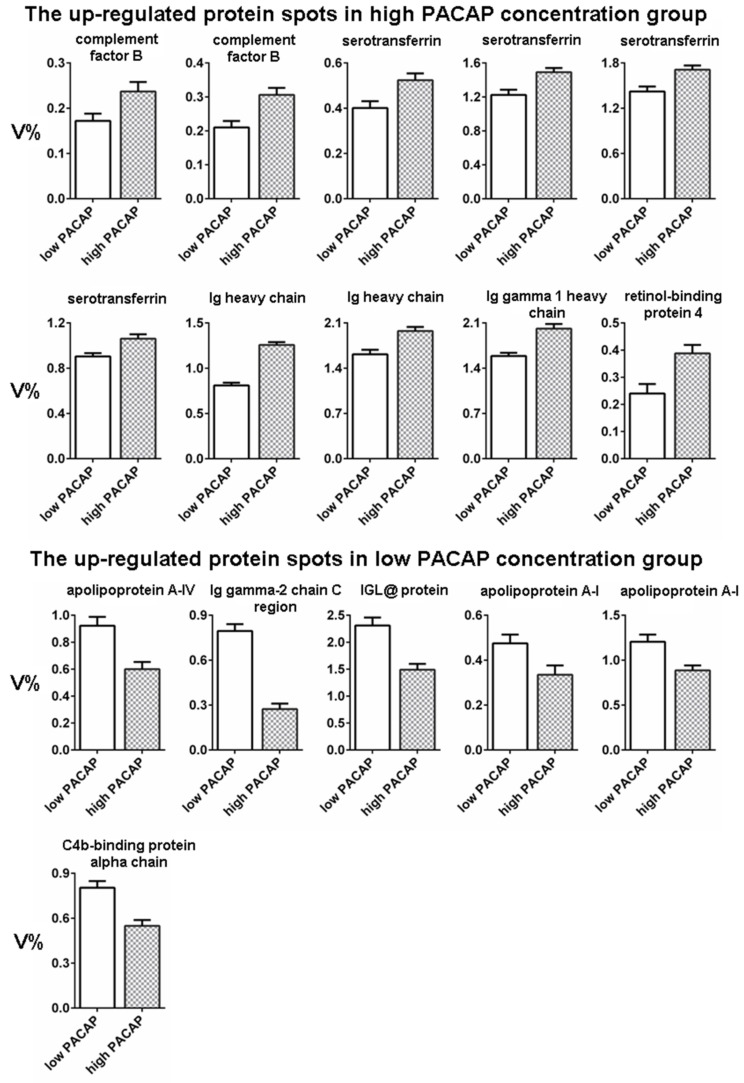
Normalized volumes (V%) of spots with statistically significant (*p* < 0.05) intensity difference between the low− and high−PACAP (pituitary adenylate cyclase-activating polypeptide) −concentration groups. Data are presented as mean ±SEM.

Ten spots showed a higher intensity in the high-PACAP-concentration group compared to six spots in the low-PACAP-concentration group (Figure 3). The 16 spots with statistically significant intensities between groups were cut out from the gels. The proteins present in the spots were identified using the LC-MS/MS method (Table 1) and classified as immune-related proteins, lipid-metabolism-related proteins and transport proteins.

**Figure 4:**
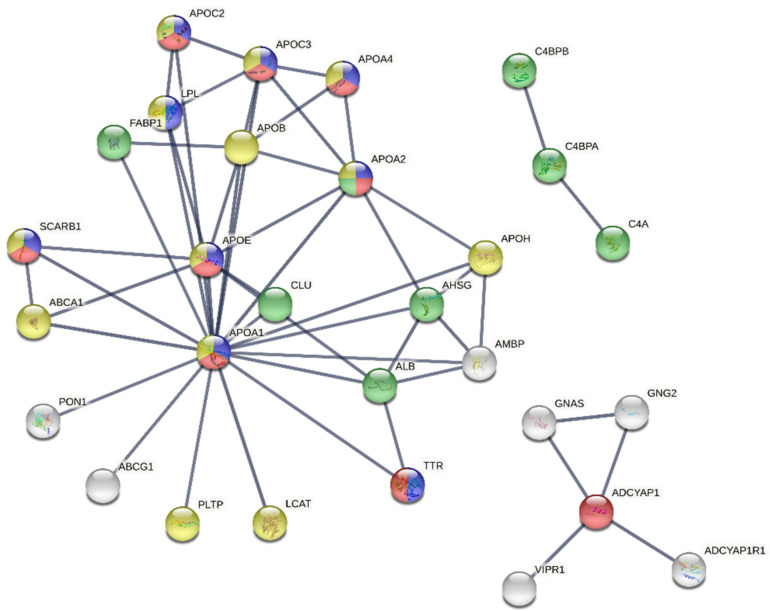
The protein–protein interaction network corresponding to the low-PACAP (pituitary adenylate cyclase-activating polypeptide)-concentration group. Colour indicates proteins take part in lipid metabolic process (blue), transport (red), response to stress (green) and cholesterol metabolism (yellow). White colour indicates the second shell of interactors. Our input: ADCYAP1: pituitary adenylate cyclase-activating polypeptide; APOA1: apolipoprotein A-I; APOA4: apolipoprotein A-IV; C4BPA: C4b-binding protein alpha chain.

**Figure 5:**
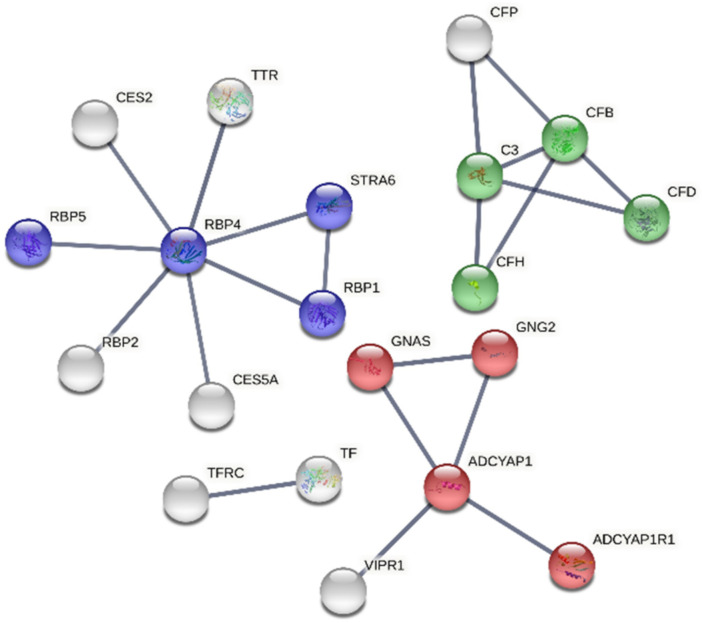
The protein–protein interaction network of proteins corresponding to the high-PACAP (pituitary adenylate cyclase-activating polypeptide)-concentration group. Colour indicates proteins take part in retinol binding (blue), adenylate cyclase-modulating G protein-coupled receptor signalling pathway (red), complement activation and alternative pathway (green). White colour indicates the second shell of interactors. Our input: ADCYAP1: pituitary adenylate cyclase-activating polypeptide; CFB: complement factor B; RBP4: retinol-binding protein 4; TF: transferrin.

The String database was used to generate the protein–protein interaction networks of the identified proteins. The bovine immunoglobulins were not found in the database, so they were omitted from the further analyses, and the interaction networks of proteins in the low- and the high-PACAP-concentration groups, respectively, plus PACAP, were generated. In both cases, the networks had very few proteins, so for the generation of the networks, the first shell of interactors was considered. The network corresponding to the low-PACAP-concentration group showed 29 proteins involved mainly in lipid metabolism, cholesterol metabolism, transport and response to stress (Figure 4). The network of proteins corresponding to the high-PACAP-concentration group contained fewer proteins (20), and the enriched functions were the complement cascade activation, retinol binding and the adenylate cyclase-modulating G protein-coupled receptor signalling (Figure 5). In case of both networks, the PACAP (ADYCAP) was not linked to the query proteins; it formed its own subnetwork, which was present in the cluster of its interaction partner proteins.

## 4. Discussion

The aim of the present study was to compare plasma protein profiles of Holstein cows with high and low plasma PACAP levels. 

Some of the proteins identified in the selected spots have a role in the immune response. Complement factor B (Bf) was identified in spot 63 and 65 showing 1.4- (*p* = 0.020) and 1.5-fold (*p* = 0.002) higher expression in the high-PACAP group, respectively. Bf is a serine protease, primarily synthesized by the liver, which activates the alternative pathway of the complement system [18]. Upon activation, complement factor B is cleaved by complement factor D and C3b, resulting in the non-catalytic chain Ba and the catalytic chain Bb. The Bb fragment enhances B-lymphocyte proliferation [19], while the Ba fragment inhibits B-cell proliferation [20]. 

Up-regulation of Ig heavy chain was observed in the high-PACAP-concentration group at three different positions on the polyacrylamide gels, showing 1.3-fold higher expression in spots 145 and 152 (*p* < 0.000) and, in spot 150, 1.2-fold (*p* = 0.004) higher expression. Contrary to Ig heavy chains, Ig gamma-2 chain C region protein in spot 260 and IGL@protein in spot 280 showed higher expression (*p* < 0.000) in the low-PACAP-concentration group. Immunoglobulins are produced by B cells to identify and neutralize pathogens. 

In spot 310, C4b-binding protein alpha chain was identified, which is mainly produced in the liver. C4b-binding glycoprotein (C4BP) is the most important soluble inhibitor of the classical and lectin pathways of the complement system [21]. The complement system is one of the key components of the innate immune system. C4BP contains seven identical alpha chains and one unique beta chain and binds vitamin K-dependent anticoagulant protein S, thereby linking the complement system with the blood coagulation [22,23]. Overexpression of C4BP was detected in cows with clinical mastitis, associated with the control of the elevated activation of the complement system [24]. The intensity of protein spot 310 was approximately 50% higher (*p* < 0.000) in the high-PACAP-concentration group. 

The other class of identified proteins belong to lipid-binding apolipoproteins. They regulate lipoprotein metabolism and transfer and redistribution of lipids among tissues [25]. Apolipoprotein A-I (apoA1) was identified in spots 285 and 287; the intensity of both spots was approximately 40% higher (*p* = 0.019 and 0.003) in the low-PACAP-concentration group. ApoA1 plays a role in cellular cholesterol efflux, and it has antioxidant and anti-inflammatory effects [26,27]. Ontsouka and his colleagues found the importance of the apoA-I/ABCA1 (ATP-binding cassette transporter) pathway in mammary gland cholesterol transport and its role in influencing milk quality and directing cholesterol back into the bloodstream [28]. Apolipoprotein A-IV (in spot 184) had 1.5-fold higher (*p* = 0.003) intensity in the low-PACAP-concentration group. Apolipoprotein A-IV (apoA4) acts primarily in intestinal lipid absorption; fat absorption increases its synthesis and secretion by the small intestine [29]. ApoA4 may be involved in short-term and long-term regulation of food intake and regulation of body weight gain [30]. Wang and his colleagues [31] also demonstrated a potential role of apoA4 in regulating glucose homeostasis. Besides apoA1, apoA4 is also a cofactor for lecithin cholesterol acyltransferase. The apolipoproteins could be secreted from blood to milk by transcytotic pathways in mammary epithelial cells, and Monks et al. concluded they may play a role in milk lipid transport in the mammary gland [32].

Besides the proteins with a role in immune functions or lipid metabolism, proteins with transport functions have also been identified. Four protein spots (103, 105, 107, 124) contained serotransferrin. These spots were located at different pI positions, which might be the consequence of post-translational modifications influencing the charge and size of the protein. The expression of each spot was higher (*p* = 0.008, *p* = 0.002, *p* = 0.002, *p* = 0.003) in the high-PACAP-concentration group. Serotransferrin is an abundant single-chain glycoprotein in plasma which is synthetized in the liver. Its main function is to bind and transport iron, but it plays an important role in many other physiological processes, such as: stimulation of cell proliferation [33], antibacterial defence [34], insulin antagonism [35], etc.

Retinol-binding protein 4 (in spot 299) had 1.6-fold higher (*p* = 0.003) expression in the high-PACAP-concentration group. The main function of the retinol-binding protein 4 (RBP4) is transporting retinol from the liver to the peripheral tissues. The vitamin A deficiency blocks its secretion post-translationally [36]. RBP4 was reported to be a fat-derived adipokine [37], inducing insulin resistance in the liver and skeletal muscle [38]. The obese diabetic rodents and humans showed increased expression of RBP4 protein, which may provide a link between insulin resistance and obesity [39].

Our results indicate that PACAP concentration is related to the lipid metabolism and immune status of cattle. Previous studies have demonstrated that the pituitary adenylate cyclase-activating polypeptide plays an important role in lipolysis and lipogenesis, as well [40]. In rat adipocytes, PACAP38 induces lipolysis by activating protein kinase A, which may phosphorylate the hormone-sensitive lipase [41]. PACAP38 also enhances isoproterenol-induced lipolysis [42]. In the presence of insulin, PACAP38 no longer has an effect on lipolysis but does impact lipogenesis [43]. In PACAP-treated adipocytes, the expression of apolipoprotein D protein and mRNA were up-regulated [39]. In our study, higher intensity of apolipoprotein A-I and apolipoprotein A-IV in the low-PACAP-concentration group was detected. 

The existence of cross-talk between the neuroendocrine and immune system is established through mediators such as PACAP which exhibit potent immunoregulatory properties. PACAP has the ability to modulate the innate and adaptive inflammatory responses at multiple sites [44]. In the high-PACAP-concentration group, some of the immune-related protein-containing spots (immunoglobulins, complement system components) showed higher intensity, providing further evidence for the immunoregulatory properties of PACAP. Lugo and her colleagues [45] have observed that PACAP could stimulate lysozyme production in fish, and through its own expression and the expression of PACAP receptors on immune cells, lysozyme can activate the complement cascade. Similarly to lysozyme, serotransferrin can be considered as a protein with antimicrobial properties providing defense against potential pathogens [46]. The level of serotransferrin was high in the high-PACAP-concentration group. 

The higher level of immune-system- and defense-related proteins in the high-PACAP-concentration group indicates the involvement of the PACAP in the regulation of the immune functions.

At the same time, in the low-PACAP-concentration group, the spots containing proteins with a role mainly in lipid and lipoprotein metabolism showed higher intensity. It is very likely that a PACAP has a complex regulatory mechanism in the adaptive and innate immune system, as spots containing proteins with a role in the immune system showed higher intensity in both groups. Based on the generated protein–protein interaction networks, it is very likely that the proteome-level changes observed in this study are not the result of the direct effect of PACAP on these proteins. Considering that PACAP is a hormone acting on transmembrane receptors, its functions point beyond direct protein–protein interactions and can have broader effects modulated by the initiated signal transduction pathways.

Our study provides useful information on the proteome-wide changes in cow plasma elicited by PACAP, and in order to deepen our knowledge, further studies are needed, such as the comparative analysis on PACAP-injected vs. noninjected cows to evaluate the changes in proteome.

## 5. Conclusions

The results of two-dimensional gel electrophoresis followed by a mass spectrometry proteomic approach obtained from the present study indicate that the concentration of pituitary adenylate cyclase-activating polypeptide in the blood plasma is closely related to the lipid metabolism and immune status of dairy cows.

## Figures and Tables

**Table 1:** Proteins identified by LC-MS/MS in spots showing statistically significant intensity difference (*p* < 0.05) between low- and high-PACAP (pituitary adenylate cyclase-activating polypeptide) -concentration groups.

Spot Number	Name of Identified Protein	Accession Number	N *	pI/Mw (Da) ^†^	Ratio ^‡^	*p*-Value
Immune-related proteins						
63	complement factor B precursor	P81187 (Bos taurus)	3	7.9/85,312	1.4	0.020
65	complement factor B precursor	P81187 (Bos taurus)	3	7.9/85,312	1.5	0.002
145	Ig heavy chain precursor	S22080 (Bos taurus)	3	6.1/50,593	1.3	0.000
150	Ig heavy chain precursor	S22080 (Bos taurus)	5	6.1/50,593	1.2	0.004
152	immunoglobulin gamma 1 heavy chain constant region	ABE68619 (Bos taurus)	2	6.5/35,878	1.3	0.000
260	Ig gamma-2 chain C region	S06611 (Bos taurus)	4	8.0/36,020	0.3	0.000
280	IGL@ protein	Q3T101 (Bos taurus)	3	5.8/24,625	0.6	0.000
310	C4b-binding protein alpha chain	Q28065 (Bos taurus)	4	6.3/21.766	0.7	0.000
Lipid-metabolism-related proteins						
184	apolipoprotein A-IV precursor	Q32PJ2 (Bos taurus)	9	5.3/42,991	0.6	0.001
285	apolipoprotein A-I	P15497 (Bos taurus)	9	5.6/28.415	0.7	0.019
287	apolipoprotein A-I	P15497 (Bos taurus)	6	5.6/28.415	0.7	0.003
Transport proteins						
103	serotransferrin precursor	Q29443 (Bos taurus)	6	6.8/77,689	1.3	0.008
105	serotransferrin precursor	Q29443 (Bos taurus)	5	6.8/77,689	1.2	0.002
107	serotransferrin precursor	Q29443 (Bos taurus)	16	6.8/77,689	1.2	0.002
124	serotransferrin precursor	Q29443 (Bos taurus)	5	6.8/77,689	1.2	0.003
299	retinol-binding protein 4	P18902 (Bos taurus)	3	5.4/21,055	1.6	0.003

* Number of unique matched peptides; ^†^ theoretical isoelectric point and molecular weight according to Expasy Swiss Bioinformatics Resource Portal; ^‡^ ratio of the protein expression of the high-PACAP-concentration group compared to the low-PACAP-concentration group.

## Data Availability

The datasets used and analyzed during the current study are available from the corresponding author on reasonable request.
